# The Local Brain Abnormalities in Patients With Transient Ischemic Attack: A Resting-State fMRI Study

**DOI:** 10.3389/fnins.2019.00024

**Published:** 2019-01-31

**Authors:** Yating Lv, Lingyu Li, Yulin Song, Yu Han, Chengshu Zhou, Dan Zhou, Fuding Zhang, Qiming Xue, Jinling Liu, Lijuan Zhao, Cairong Zhang, Xiujie Han

**Affiliations:** ^1^Institutes of Psychological Sciences, Hangzhou Normal University, Hangzhou, China; ^2^Zhejiang Key Laboratory for Research in Assessment of Cognitive Impairments, Hangzhou, China; ^3^Department of Neurology, Anshan Changda Hospital, Anshan, China; ^4^Department of Neurology, The First Affiliated Hospital, Dalian Medical University, Dalian, China; ^5^Department of Image, Anshan Changda Hospital, Anshan, China; ^6^Department of Ultrasonics, Anshan Changda Hospital, Anshan, China

**Keywords:** resting-state fMRI, transient ischemic attack, amplitude of low frequency fluctuation, regional homogeneity, degree centrality

## Abstract

**Background:** Transient ischemic attack (TIA) is an important risk factor for stroke. Despite the transient episodes of clinical symptoms, brain alterations are still observed in patients with TIA. However, the functional mechanism of transient ischemia is still unclear. Here, we employed resting-state functional magnetic resonance imaging (rs-fMRI) to explore the functional abnormalities in patients with TIA.

**Methods:** 48 TIA patients and 41 age- and sex-matched healthy controls (HCs) were enrolled in the study. For each participant, we collected rs-fMRI data and clinical/physiological/biochemical data. Amplitude of low frequency fluctuation (ALFF), regional homogeneity (ReHo), and degree centrality (DC) were then calculated. Two sample *t*-tests were performed to compare the ALFF, ReHo, and DC maps between the two groups. Furthermore, a correlation analysis was performed to explore the relationship between local brain abnormalities and clinical/physiological/biochemical characteristics tests in TIA patients.

**Results:** Compared with the HCs, the TIA patients exhibited decreased ALFF in the left middle temporal gyrus, decreased DC in the triangular part of right inferior frontal gyrus, and no significant statistical difference in ReHo. No correlation was found between local abnormalities and clinical/physiological/biochemical scores in the patients with TIA.

**Conclusion:** Collectively, we found decreased ALFF and DC in patients with TIA which provide evidence for local brain dysfunctions and may help to understand the pathological mechanism for the disease.

## Introduction

Transient ischemic attack (TIA) is an episode of reversible temporary neurologic dysfunction caused by focal cerebral ischemia of the brain (Albers et al., 2002; Easton et al., 2009). Despite the transient episodes of clinical symptoms, structural and functional brain alterations are still observed in patients with TIA. For example, using structural MRI, one previous study reported that patients with TIA exhibited gray matter (GM) atrophy in specific regions of the default mode network (Li et al., 2015). Functionally, based on arterial spin labeling (ASL) MRI, several studies identified TIA-related perfusion deficits as characterized by decreased cerebral blood flow (CBF) in widespread brain regions (MacIntosh et al., 2010; Kleinman et al., 2012; Zaharchuk et al., 2012; Qiao et al., 2013). Moreover, TIA is an important risk factor for eventual stroke or a silent stroke (Giles and Rothwell, 2007; Easton et al., 2009), and thus represents a key time window for early diagnosis and intervention of stroke. However, the local brain functional mechanism of transient ischemia still unclear.

Resting-state functional magnetic resonance imaging (rs-fMRI) is a promising tool to investigate functional alterations of the human brain, which has unique advantages in clinical conditions because it does not require participants to engage in cognitive activities (Biswal et al., 1995; Fox and Raichle, 2007). Although the majority of analytic techniques [functional connectivity (FC), graph theory, independent component analysis (ICA), etc] for rs-fMRI data characterize the function of brain network, the local dynamics cannot be fully addressed with these approaches. Recently, several methods have been proposed to characterize the local properties of the rs-fMRI signal: amplitude of low frequency fluctuation (ALFF) (Zang et al., 2007), regional homogeneity (ReHo) (Zang et al., 2004), and degree centrality (DC) (Buckner et al., 2009).

ALFF is defined as the mean amplitude of fluctuations within low frequency range. It provides direct characterization to spontaneous brain activity at each voxel (Zang et al., 2007; Zuo et al., 2010). ReHo is proposed as a voxel-wise measure of the synchronization of the time courses of neighboring voxels based on the hypothesis that voxels within a functional brain area synchronize their metabolic activity depending on specific conditions (Zang et al., 2004). While DC is proposed to map the degree of intrinsic FC across the brain in order to reflect a stable property of cortical network architecture at the voxel level (Buckner et al., 2009). The three local metrics have been widely utilized to investigate functional modulations in many neuropsychiatric disorders (Liu et al., 2006; Zang et al., 2007; Wu et al., 2009; Hoptman et al., 2010; Paakki et al., 2010; Liang et al., 2011; Premi et al., 2014; Zhao et al., 2014; Dai et al., 2015). Specifically, in patients with brain ischemia, several research groups have reported local functional alterations (Guo et al., 2014; Tsai et al., 2014; Shi et al., 2017). For example, Tsai and colleagues reported decreased ALFF in precuneus and posterior cingulate cortex regions in acute stroke patients as compared with healthy controls (HCs) (Tsai et al., 2014).

The three voxel-wised metrics define brain functional characteristics from different perspectives and present the progressive relationship. For a single voxel, ALFF characterizes neural activity intensity of this voxel, ReHo reveals the importance of this voxel among the nearest voxels, while DC portrays the importance of this voxel in the whole brain. Regional abnormalities could be identified with greater sensitivity by applying these three metrics. For example, An and colleagues showed the group differences of ADHD patients and HCs using both ALFF and ReHo, they observed that regions exhibiting group differences in ReHo and ALFF metrics were not completely the same (An et al., 2013), which suggest that these metrics complement each other and characterize local brain abnormalities from different perspectives.

In the current study, we employed rs-fMRI to explore the local abnormalities in patients with TIA from different perspectives. Specifically, we sought to determine whether and how TIA disrupts the local function using three local metrics (ALFF, ReHo, and DC) and whether those local abnormalities (if observed) are associated with clinical/physiological/biochemical characteristics scores of the patients.

## Materials and Methods

### Participants

From April 2015 to June 2016, 51 suspected TIA patients who had transient neurologic symptoms which had been evaluated to have a possible vascular etiology judged by clinical neurologists were recruited from Department of Neurology, Anshan Changda Hospital. Patients with hemorrhage, leukoaraiosis, migraine, epilepsy or psychiatric diseases history were excluded. All patients underwent electrocardiogram (ECG), carotid duplex ultrasound examination (CDU) and MRI scan. The study was approved by the Ethics Committee of the Center for Cognition and Brain Disorders, Hangzhou Normal University. Written informed consent was obtained from all participants.

For each patient, we recorded information as follows: (1) history of TIA and stroke; (2) previous risk factors: hypertension, diabetes mellitus, coronary artery disease, current smoking and drinking; (3) medications used before the MRI scanning; (4) in-hospital evaluation of arterial stenosis (carotid duplex ultrasound and MR angiography), atrial fibrillation (ECG) and brain infarcts (diffusion-weighted imaging and T2-FLAIR); (5) one-year telephone follow-up of stroke and/or TIA attack. Notably, four patients dropped out in one-year follow-up. Based on the methods described by Johnston et al. (2007), an ABCD2 score was generated for each patient to evaluate the risk for subsequent stroke.

41 age- and sex-matched HCs with no physical diseases or history of psychiatric or neurologic disorders from local community were also recruited in this study.

Three patients were excluded from the final analysis due to image quality of multimodal MRI (see below for details), leaving 48 TIA patients and 41 HCs in the final analysis. Out of the 48 patients, 4 (8.3%) experienced stroke, 25 (52.1%) experienced TIA, and 23 (47.9%) were first episode. Detailed demographic and clinical information for all participants are summarized in Table 1.

**Table 1:** Demographics and clinical characteristics of all participants.

	TIA	HCs	
	(n = 48)	(n = 41)	*p*-value
Age (years)	57.604 ± 9.778	55.024 ± 8.033	0.182^a^
Sex (M/F)	37/11	30/11	0.670^b^
MMSE	29.208 ± 2.609	28.615 ± 1.664	0.222^a^
Blood systolic pressure (mmHg)	145.542 ± 20.753	126.940 ± 19.758^c^	<0.001^a^
Blood diastolic pressure (mmHg)	86.667 ± 10.383	80.030 ± 10.896^c^	0.007^a^
Blood sugar level (mmol/L)	6.299 ± 2.113	5.200 ± 0.740^c^	<0.001^a^
Total cholesterol (mmol/L)	5.275 ± 1.173	4.753 ± 1.011^c^	0.037^a^
Triglycerides (mmol/L)	1.603 ± 0.940	1.917 ± 1.345^c^	0.234^a^
HDL-C (mmol/L)	1.111 ± 0.238	1.051 ± 0.290^c^	0.311^a^
LDL-C (mmol/L)	3.314 ± 0.974	2.691 ± 0.904^c^	0.004^a^
ABCD2 scores, median	4 (2–6)	–	–
Smoking, No. (%)	31 (64.6%)	19 (46.3%)	0.084^b^
Drinking, No. (%)	20 (41.7%)	21 (51.2%)	0.367^b^
Hypertension, No. (%)	22 (45.8%)	6 (14.6%)	0.002^b^
Diabetes, No. (%)	8 (16.7%)	0 (0%)	0.006^b^
Coronary artery disease, No. (%)	2 (4.2%)	0 (0%)	0.186^b^
Atrial fibrillation, No. (%)	1 (2.1%)	–	–
Medication	–	–	–
Antiplatelets, No. (%)	48 (100%)	–	–
Statins, No. (%)	2 (4.2%)	–	–
DWI hyperintensity, No. (%)	6 (12.5%)	–	–
Vessel stenosis, No. (%)	9 (18.8%)	–	–
TIA/stroke attack in one-year follow-up, No. (%)	12 (27.3%)^d^	–	–

*TIA, transient ischemic attack; HCs, healthy controls; M, male; F, female; HDL-C, high-density lipoprotein cholesterol; LDL-C, low-density lipoprotein cholesterol; MMSE, mini-mental state examination; DWI, diffusion weighted imaging. ^*a*^Data were obtained using two-sample two-side t-tests. ^*b*^Data were obtained using Pearson Chi-square tests. ^*c*^Data were missing for six controls. ^*d*^Four patients dropped out in the one-year follow-up*.

### Physiological and Biochemical Tests

All participants completed a series of physiological/biochemical tests within 24 h before the MRI data acquisition, including blood systolic pressure, blood diastolic pressure, blood sugar level, total cholesterol, triglycerides, high-density lipoprotein cholesterol (HDL-C), and low-density lipoprotein cholesterol (LDL-C). Additionally, all participants underwent the mini-mental state examination (MMSE) to evaluate global cognition (Schultz-Larsen et al., 2007).

### MR Data Acquisition

MR data was acquired using a GE MR-750 3.0 T scanner (GE Medical Systems, Inc., Waukesha, WI, United States) at Anshan Changda Hospital, China. The time interval between the last TIA attack and subsequent MRI scanning was 0.25–6 days for the patients. During the data acquisition, participants were instructed to keep awake, relax with their eyes closed and remain motionless as much as possible.

Resting-state fMRI (rs-fMRI) data was obtained using an echo-planar imaging sequence with following protocols: 43 axial slices, TR = 2000 ms, TE = 30 ms, flip angle = 60°, matrix = 64 × 64, in-plane resolution of 3.44 mm × 3.44 mm, thickness/gap = 3.2/0 mm, 240 contiguous EPI functional volumes, 8 min.

3D high resolution T1-weighted anatomical images were acquired using a 3D-MPRAGE sequence: 176 sagittal slice, TR = 8100 ms, TE = 3.1 ms, matrix = 256 × 256, voxel size: 1 mm × 1 mm × 1 mm, thickness/gap = 1/0 mm. This session lasted for about 5 min.

Three patients were excluded from further analysis due to incomplete coverage of the whole brain for rs-fMRI scan (2) or the lost of 3D T1 image (1).

### Data Preprocessing

Resting-state fMRI data was processed using Data Processing & Analysis for Brain Imaging (DPABI) (Yan et al., 2016) including: (1) removing first 10 time points to make the longitudinal magnetization reach steady state and to let the participant get used to the scanning environment; (2) slice-timing to correct the differences in image acquisition time between slices; (3) head motion correction; (4) spatial normalization to the Montreal Neurological Institute (MNI) space via the deformation fields derived from tissue segmentation of structural images (resampling voxel size = 3 mm × 3 mm × 3 mm); (5) spatial smoothing with an isotropic Gaussian kernel with a full width at half maximum (FWHM) of 6 mm; (6) removing linear trend of the time course; (7) regressing out the head motion effect (using Friston 24 parameter) from the fMRI data (Friston et al., 1996); (8) band-pass filtering (0.01–0.08 Hz). No participants were excluded from further analysis due to large head motion (more than 3.0 mm of maximal translation in any direction of *x*, *y* or *z* or 3.0° of maximal rotation throughout the course of scanning). Then, 3 voxel-wise whole-brain analytic methods, i.e., ALFF, ReHo, and DC, were further applied to the preprocessed fMRI data.

### ALFF Calculation

After data preprocessing, the time course for each voxel was transformed to the frequency domain with a fast Fourier transform and the power spectrum was then obtained. The square root was calculated at each frequency of the power spectrum and the averaged square root was obtained across 0.01–0.08 Hz at each voxel as the ALFF value, which was further divided by the global mean ALFF of each individual for group comparison (Zang et al., 2007).

### ReHo Calculation

The Kendall’s coefficient of concordance (KCC) was used to measure the local synchronization of the time series of neighboring voxels as follows (Zang et al., 2004):

(1) $$W=\frac{{\sum {\left(R_{i}\right)}^{2}-n\left(\bar{R}\right)}^{2}}{\frac{1}{12}K^{2}\left(n^{3}-n\right)}$$

where *W* is the KCC among given voxels, ranged from 0 to 1; *R_i_* is the sum rank of the *i*th time point; 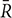 = (n+1)*K*)/2 is the mean of the *R_i_*’s; K is the number of time series within a measured cluster (K = 7, 19, and 27, respectively. 27 in the current study); *n* is the number of ranks. The ReHo value of each voxel was then divided by the global mean ReHo of each individual for standardization purposes. Note that the spatial smoothing (FWHM = 6 mm) was performed after ReHo calculation.

### DC Calculation

Several nuisance signals (white matter, cerebrospinal fluid, and global mean signal) were further regressed out from each voxel’s time series. For a weighted graph, DC is defined as the sum of weights from edges connecting to a node (also sometimes referred to as the node strength) (Zuo et al., 2012). Pearson’s correlation of time series was performed between each voxel and every other voxel in the entire brain to calculate a correlation matrix R = (r*_ij_*), *j* = 1...*N* (*N* is the number of voxels), *i* ≠ 1 (Buckner et al., 2009; Zuo et al., 2012). The correlation coefficients with r*_ij_* ≥ 0.32 (*p* < 0.05, Bonferroni-corrected over whole-brain voxels) were summed up for each voxel and then a weighted DC was obtained for each voxel. The threshold was used to eliminate counting voxels that had low temporal correlation (Buckner et al., 2009).

The weighted DC of each voxel was further divided by the global mean weighted DC of each individual for group comparison.

### Statistical Analysis

The age, clinical/physiological/biochemical variables were analyzed with the Statistical Package for the Social Sciences (SPSS) (SPSS Inc., Chicago, IL, United States). The differences between the patients and the HCs in age, clinical/physiological/biochemical tests were tested with Student’s *t*-tests. Sex difference was tested with the Pearson Chi-Square test.

Two sample *t*-tests were performed to compare the ALFF, ReHo, and DC maps between patients with TIA and HCs respectively. Individual age and sex were treated as covariates during the group comparisons to minimize their potential effects on our results. The resultant T-maps were thresholded with voxel *p* < 0.001, cluster *p* < 0.05 (Gaussian Random Field theory (GRF) correction for multiple comparisons). The analyses were performed using DPABI (Yan et al., 2016).

For any measure (ALFF, ReHo, or DC) showing TIA-related alterations, a Pearson correlation analysis was used to assess its associations with clinical/physiological/biochemical characteristics of the patients (including blood systolic pressure, blood diastolic pressure, blood sugar level, total cholesterol, triglycerides, HDL-C, LDL-C). The correlations were considered significant at a threshold of *p* < 0.05.

## Results

### Participants’ Characteristics

As shown in Table 1, there were no significant differences in sex (*p* = 0.670), age (*p* = 0.182), MMSE scores (*p* = 0.222), smoking (*p* = 0.084), drinking (*p* = 0.367), or coronary artery disease (*p* = 0.186) between TIA patients and HCs. Hypertension (*p* = 0.002) and diabetes (*p* = 0.006) showed significant between-group differences. Significantly higher blood systolic pressure (*p* < 0.001), diastolic pressure (*p* = 0.007), blood sugar level (*p* < 0.001), total cholesterol (*p* = 0.037) and LDL-C (*p* = 0.004) were observed in the patients compared with the HCs. The median ABCD2 score for the patients with TIA was four (Table 1).

### Disrupted Local Function in TIA

**Figure 1:**
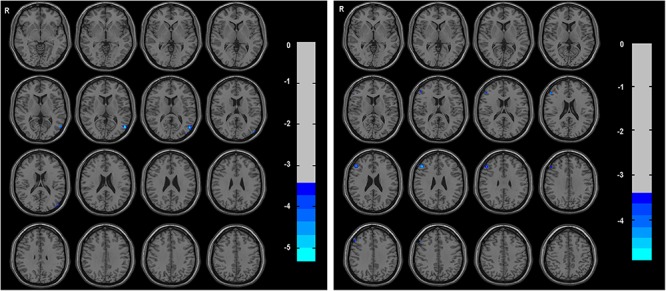
The group differences of ALFF (left) and DC (right) between TIA patients and healthy controls, respectively. Cold colors indicate decreased ALFF in left middle temporal gyrus and decreased DC in triangular part of right inferior frontal gyrus in patients with TIA as compared to that in healthy controls (voxel *p* < 0.001, cluster *p* < 0.05, GRF correction, set cluster size >31 voxels for ALFF metric and cluster size >28 voxels for DC metric).

Compared with HCs, the TIA patients exhibited decreased ALFF in the left middle temporal gyrus (voxel *p* < 0.001, cluster *p* < 0.05, GRF correction, cluster size >31 voxels) (Table 2 and Figure 1).

**Table 2:** Regions showing abnormal ALFF and DC in patients with TIA as compared with HCs.

			MNI coordinate (mm)		
Regions	Cluster size	Peak T value	X	Y	Z
**ALFF**
Left middle temporal gyrus	40	-4.869	-51	-66	9
**DC**
Triangular part of right inferior frontal gyrus	56	-4.557	48	33	27

The triangular part of right inferior frontal gyrus showed decreased DC in TIA patients as compared with HCs (voxel *p* < 0.001, cluster *p* < 0.05, GRF correction, cluster size >28 voxels) (Table 2 and Figure 1).

No regions showed significant between-group differences in ReHo (voxel *p* < 0.001, cluster *p* < 0.05, GRF correction, cluster size >78 voxels).

### Relationship Between Local Metrics and Clinical/Physiological/Biochemical Characteristics

No significant correlation was found between local brain abnormalities and clinical/physiological/biochemical characteristics in TIA patients (*p* > 0.05) (Table 3).

**Table 3:** Correlation between local metrics and clinical/physiological/ biochemical characteristics.

	ALFF	DC
Clinical/physiological/ biochemical characteristics	Left middle temporal gyrus	Triangular part of right inferior frontal gyrus
Blood systolic pressure	*r* = -0.050 *p* = 0.736	*r* = 0.256 *p* = 0.069
Blood diastolic pressure	*r* = -0.167 *p* = 0.256	*r* = 0.043 *p* = 0.771
Blood sugar level	*r* = 0.218 *p* = 0.136	*r* = -0.011 *p* = 0.941
Total cholesterol	*r* = -0.046 *p* = 0.756	*r* = 0.075 *p* = 0.614
Triglycerides	*r* = -0.125 *p* = 0.397	*r* = -0.062 *p* = 0.673
HDL-C	*r* = 0.030 *p* = 0.841	*r* = -0.170 *p* = 0.248
LDL-C	*r* = -0.042 *p* = 0.778	*r* = 0.164 *p* = 0.266
MMSE	*r* = -0.213 *p* = 0.147	*r* = -0.280 *p* = 0.054

*HDL-C, high-density lipoprotein cholesterol; LDL-C, low-density lipoprotein cholesterol; MMSE, mini-mental state examination*.

## Discussion

The ALFF, ReHo, and DC define brain local function from different perspectives and present the progressive relationship. Regional abnormalities could be identified with greater sensitivity by applying three metrics. In this study, we used these three rs-fMRI analysis metrics to investigate local brain functional alterations in patients with TIA and further examined the relevance of these alterations induced by brain ischemia with respect to clinical/physiological/biochemical characteristics scores. Our results showed that compared with the HCs, the TIA patients exhibited decreased ALFF in the left middle temporal gyrus, decreased DC in the triangular part of right inferior frontal gyrus. These findings have implications for understanding the functional mechanisms in the early stage of brain ischemia.

ALFF was supposed to reflect the extent of spontaneous neuronal activity (Zang et al., 2007). In the present study, the left middle temporal gyrus of TIA patients showed decreased ALFF, which indicated decreased spontaneous neuronal activity within the local brain region. The middle temporal gyrus was involved in several cognitive processes, including language and semantic memory processing, as well as visual perception (Söderfeldt et al., 1997; Cabeza and Nyberg, 2000; Li et al., 2013; Bonilha et al., 2017). Previous study showed that decreased FC in left middle temporal gyrus within the default mode network in patients with TIA as compared with HCs (Li et al., 2013). Thus, we speculate that the decreased local neuronal activity (ALFF) in the left middle temporal gyrus could be the reason for the aberrant FC in TIA. Moreover, despite the transient episodes of the clinical symptoms, TIA was also accompanied by cognitive impairments in multiple domains including executive function, information processing speed and abstraction (Bakker et al., 2003; Sachdev et al., 2004). The difficulties in language processing in patients with TIA may be attributable to the decreased ALFF in left temporal gyrus.

ReHo reflects the local synchronization of spontaneous BOLD signal. The decreased ReHo indicates decreased local synchronization of low frequency fluctuations of the BOLD signal (Lv et al., 2013). When applying threshold of *p* < 0.001 (GRF correction for multiple comparisons), there was no significant difference in ReHo between TIA and HCs. While using voxel *p* < 0.05 and cluster size larger than 25 contiguous voxels, Guo and colleagues found decreased ReHo in the right dorsolateral prefrontal cortex, inferior prefrontal cortex, ventral anterior cingulate cortex, and dorsal posterior cingulate cortex in patients with TIA (Guo et al., 2014). We also found decreased ReHo in right inferior prefrontal gyrus and cingulate cortex using the same threshold (voxel *p* < 0.05, cluster size >25 voxels) as Guo et al. (additional data are given in Online Resource, Supplementary Figure S1). These findings may indicate that some true positive brain regions may not survive the multiple comparison correction when using strict threshold to decrease false positive (Eklund et al., 2016).

DC reflects the role and status of voxels in brain network and represents the most local and directly quantifiable centrality measure (Buckner et al., 2009). Here, the triangular part of right inferior frontal gyrus exhibited decreased DC, which indicated decreased importance of this region in the brain of TIA. The triangular part of right inferior frontal gyrus was involved in several cognitive processes, including attention and motor inhibition processes, as well as language performance (Hampshire et al., 2010; Tanaka et al., 2013; Hassa et al., 2016). Liu and colleagues showed decreased FC between the inferior frontal cortex and dorsal attention network in patients with post-stroke memory dysfunction (Liu et al., 2017). Moreover, the activation of the right inferior frontal gyrus may be essential for language performance in patients experiencing aphasia after left hemispheric stroke (Winhuisen et al., 2005, 2007). Thus, we speculate that the decreased DC in the triangular part of right inferior frontal gyrus may indicate the decreased FC with attention and language network, and may induce attention, inhibitory control, and language impairments in TIA.

The present study has some limitations. First, this study lacked cognitive data for the patients. It would be interesting to investigate the relationships between functional alterations and cognitive dysfunction associated with TIA. Second, we did not collect MRI data during the follow up period, and thus cannot examine how functional brain networks reorganize as TIA continues to advance. Future longitudinal studies are warranted to examine whether the current approach could be used to monitor disease progression of TIA.

## Ethics Statement

The study has been approved by the local ethics committee and has been performed in accordance with the ethical standards laid down in the 1964 Declaration of Helsinki and its later amendments. Written informed consent has been obtained from all study participants.

## Author Contributions

YL and XH designed the study. YS, YH, chZ, DZ, FZ, QX, JL, LZ, and caZ performed the experiments and collected the data. YL and LL analyzed and interpreted the data, wrote the manuscript.

## Conflict of Interest Statement

The authors declare that the research was conducted in the absence of any commercial or financial relationships that could be construed as a potential conflict of interest.
