# Supplementary figures and images for: The Local Brain Abnormalities in Patients With Transient Ischemic Attack: A Resting-State fMRI Study
Source: Front Neurosci. 2019 Jan 31;13:24. doi: 10.3389/fnins.2019.00024 (PMC6371034; doi:10.3389/fnins.2019.00024)

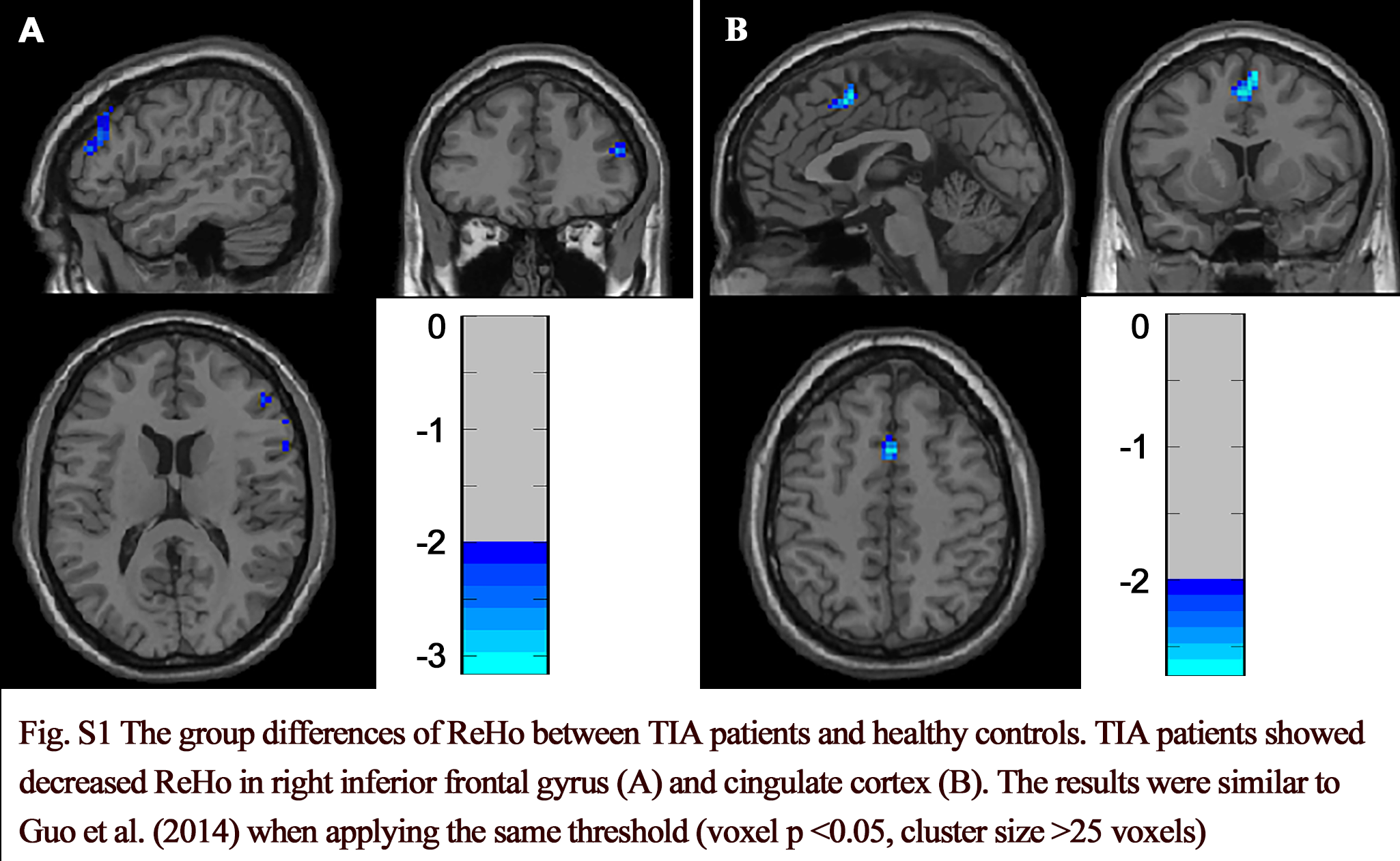

Supplement: Supplementary file 1 [file Image_1.TIF]
